# Duodenal Atresia: Open versus MIS Repair—Analysis of Our Experience over the Last 12 Years

**DOI:** 10.1155/2017/4585360

**Published:** 2017-02-23

**Authors:** Salvatore Fabio Chiarenza, Valeria Bucci, Maria Luisa Conighi, Elisa Zolpi, Lorenzo Costa, Lorella Fasoli, Cosimo Bleve

**Affiliations:** Department of Pediatric Surgery and Pediatric Minimally Invasive Surgery and New Technologies, San Bortolo Hospital, Vicenza, Italy

## Abstract

*Objective*. Duodenal atresia (DA) routinely has been corrected by laparotomy and duodenoduodenostomy with excellent long-term results. We revisited the patients with DA treated in the last 12 years (2004–2016) comparing the open and the minimally invasive surgical (MIS) approach.* Methods*. We divided our cohort of patients into two groups. Group 1 included 10 patients with CDO (2004–09) treated with open procedure: 5, DA; 3, duodenal web; 2, extrinsic obstruction. Three presented with Down's syndrome while 3 presented with concomitant malformations. Group 2 included 8 patients (2009–16): 1, web; 5, DA; 2, extrinsic obstruction. Seven were treated by MIS; 1 was treated by Endoscopy. Three presented with Down's syndrome; 3 presented with concomitant malformations.* Results*. Average operating time was 120 minutes in Group 1 and 190 minutes in Group 2. In MIS Group the visualization was excellent. We recorded no intraoperative complications, conversions, or anastomotic leakage. Feedings started on 3–7 postoperative days. Follow-up showed no evidence of stricture or obstruction. In Group 1 feedings started within 10–22 days and we have 1 postoperative obstruction.* Conclusions*. Laparoscopic repair of DA is one of the most challenging procedures among pediatric laparoscopic procedures. These patients had a shorter length of hospitalization and more rapid advancement to full feeding compared to patients undergoing the open approach. Laparoscopic repair of DA could be the preferred technique, safe, and efficacious, in the hands of experienced surgeons.

## 1. Introduction

Duodenal atresia (DA) is a fairly common congenital anomaly occurring in approximately 1 per 5000 to 10000 live births, affecting boys more commonly than girls. More than 50% of affected patients have associated congenital anomalies: trisomy 21 (approximately 30% of patients), as part of the VACTERL complex of anomalies (vertebral, anorectal, cardiac, oesophageal atresia, renal, and limb anomalies); isolated cardiac defects, 30%; prematurity 45%; growth retardation 33%; other intestinal anomalies, 25% [1, 2].

Typically, the diagnosis is made by prenatal ultrasound with a history of polyhydramnios (32% to 81%) and the detection of two fluid-filled structures consistent with a “double bubble” (the stomach and the dilated proximal duodenum), in up to 44% of case. The amniotic fluid-filled “double bubble” may represent an intrinsic or extrinsic obstruction [3]. To confirm duodenal obstruction is important to visualize the dilated duodenum for several minutes as it is possible that intestinal peristalsis in a fetus may show transient dilatation suggesting duodenal obstruction [4]. It is also important to demonstrate the continuity between the gastric and duodenal bubbles to exclude other causes. Choudhry et al. (2009) [3] showed that the prenatal diagnosis was made on prenatal ultrasound at or earlier than 20 weeks of gestation in keeping with the previously mentioned literature [3, 5, 6].

At birth, plain abdominal radiograph reveals the classic double bubble sign with no distal gas [1]. The presentation of the neonate varies depending on the following: complete or incomplete obstruction and location of Vater's ampulla in relation to the obstruction (postampullary approximately in 85%).

The first report of surgical correction of DA was by Ladd in 1931 with a reported mortality of 40% [7]. The traditional method of repair of DA is an open duodenoduodenostomy in a diamond-shaped configuration, described by Kimura et al. in 1990 [8]. This technique has become the standard.

Recent improvements in laparoscopic equipment and techniques have sparked a revolution in the surgical care of infants and children. The introduction of advanced laparoscopic techniques in the neonate has more recently led to a new surgical approach, the laparoscopic duodenoduodenostomy [1]. The first reports of laparoscopic repair of duodenal atresia date 2001 and 2002, when shortly after each other Bax et al. [9] and Rothenberg [10] described their initial experience with this approach [11]. Based on our experiences with MIS approach in neonates to treat other congenital anomalies, we elected to undertake the evaluation and treatment of patients presenting with duodenal obstruction using a laparoscopic approach. We revisited the patients with DA treated in the last 12 years comparing the open and the MIS approach.

## 2. Materials and Methods

We conducted a standardized chart review of all records from our Institution from January 2004 to January 2015. All cases with a diagnosis of “intestinal atresia” were obtained and then hand-screened to select only those cases of duodenal atresia or stenosis. All cases of congenital duodenal obstruction (CDO) seen in our Institution, Pediatric Minimally Invasive Surgery and New Technologies of San Bortolo Hospital, in Vicenza, Italy, were then reviewed.

Data collected included method of diagnosis, associated anomalies, patient age and weight at surgery, operative procedures performed, operative time, any intraoperative complications, and postoperative course.

We divided our patients into two homogeneous groups, Tables 1(a) and 1(b). Group 1 consisted of 10 patients between 2004 and 2009 treated with an open procedure until the laparoscopic approach was introduced. Of these patients, 5 had a duodenal atresia (DA), 3 a duodenal web, and 2 an extrinsic obstruction (an annular pancreas with a complete obstruction of the lumen and a preduodenal portal vein with a quite complete obstruction). Three had Down syndrome and 3 concomitant malformations. Group 2 consisted of 8 patients that underwent operation between 2009 and 2015 (December 2014). These patients were treated with MIS approach: 7 had laparoscopic procedure performed with 3 mm instruments and 1 had endoscopic web resection. This group included 5 DA, 1 duodenal web, and 2 extrinsic obstructions (both presented an annular pancreas with a complete obstruction of the lumen). Three patients had Down syndrome and 3 concomitant malformations.

**Figure 1:**
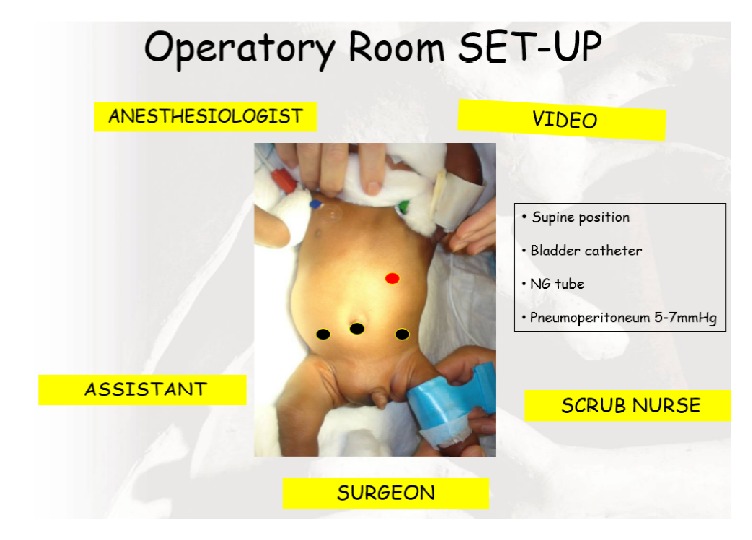
Operatory room set-up.

The operating room set-up is represented in Figure 1. The surgeon stands at the foot of the table, the first assistant/camera operator is at the foot of the table on the patient's left side to allow the surgeon performance while the scrub nurse stands on the patients right side. The monitor is positioned at the head right side of the patients while the anaesthesiologist stands at the head of the table on the left side. With the patient in the supine/semilateral position, general anesthesia is induced. The abdomen is prepared and draped in the usual sterile fashion.

The procedure began with umbilical scar incision. The dissection was carried out down through the subcutaneous tissues (open access), and the umbilical arteries and vein are dissected free and ligated. Under direct vision, a 5 mm port is placed into the peritoneal cavity. The abdomen is insufflated with carbon dioxide (5–7 mmHg, 2 l/min) and a 30° angle telescope is placed into the abdominal cavity, which is then inspected for additional anomalies (malrotation or intestinal atresia).

**Figure 2:**
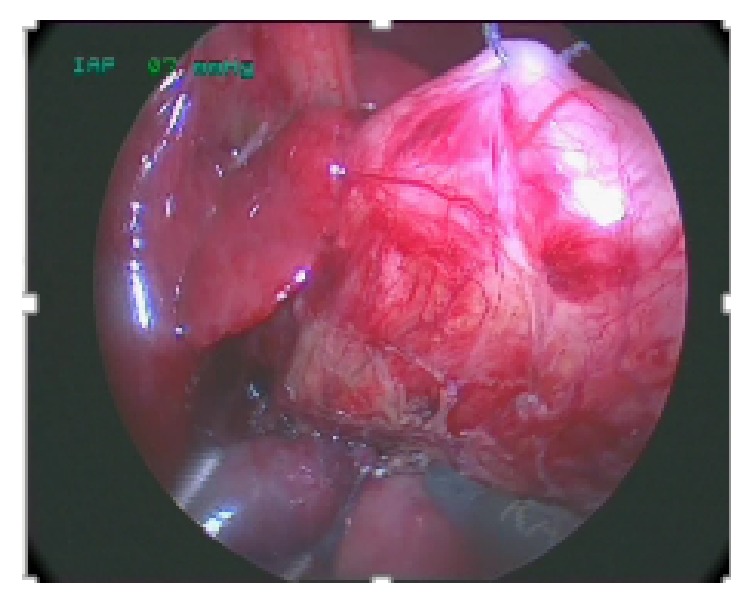
Traction suture on proximal dilated duodenum through the superior portion of this segment (serosal layer) to expose correctly the inferior surface.

**Figure 3:**
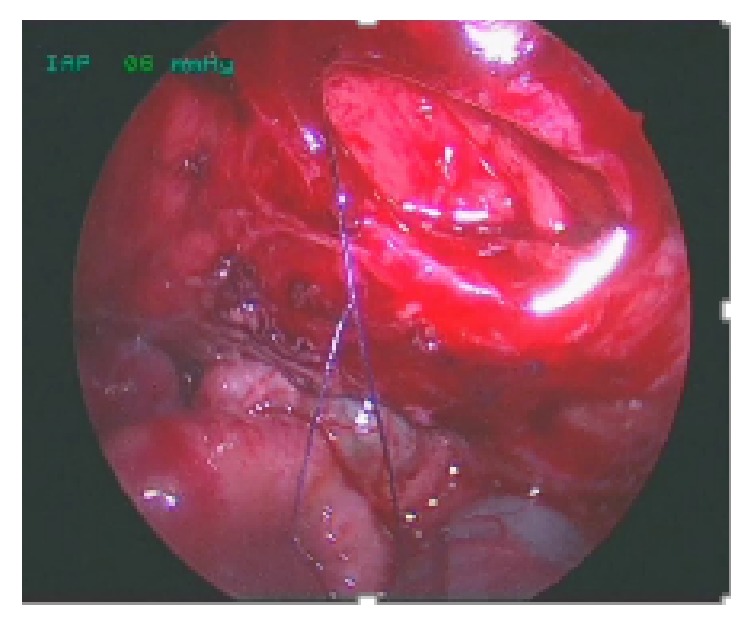
Transverse incision of proximal duodenum.

Then, two additional 3 mm trocars for 3 mm instruments were inserted under direct vision in the lower right and left quadrant. An additional 3 mm grasping forceps can be introduced in the left epigastric quadrant for lifting the liver. A personal trick consists in positioning a transcutaneous traction suture around the hepatic falciform ligament to lift up the liver avoiding the need of the 3 accessory port, Figure 2. In this way, we gain access to the area of the bulbus duodeni. The transverse colon (gastrocolic ligament) is partially dissected from the stomach and duodenum and reflected inferiorly. The duodenum is then mobilized from its retroperitoneal position and the dilated proximal duodenal atresic end is identified. At this point, we introduce one stay suture transcutaneously through the superior portion of this segment (serosal layer) to expose correctly the inferior surface making a transverse incision, Figure 3. The second and third portions of the duodenum are adequately mobilized using a “no touch” technique as much as possible to allow a tension-free diamond-shaped duodenoduodenostomy.

If there are some doubts regarding the incomplete atresia (internal duodenal web) we introduce and gently push a nasogastric tube down toward the distal part of the duodenum to check a possible internal obstruction. In this case pushing the tube we can clearly detect an incisure on the duodenal surface.

**Figure 4:**
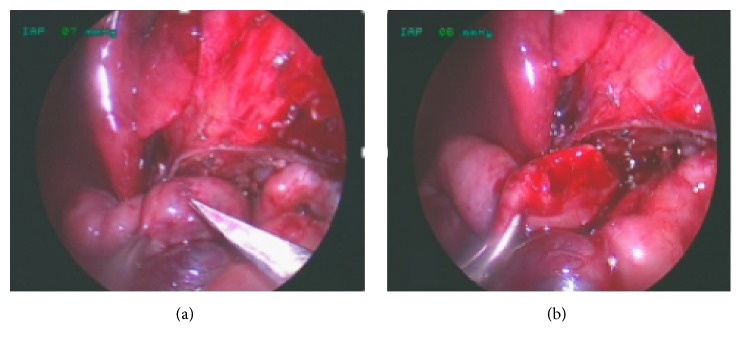
(a) Distal atresic duodenum; (b) longitudinal incision of superior surface of distal duodenum.

The second surgical step is to incise distal duodenum longitudinally with scissors and open the bulbus at a convenient place transversely for easy anastomosis, Figures 4(a) and 4(b). In case of internal obstruction a longitudinal incision along the proximal delineated insertion of the web is performed down to the distal duodenum and the occlusive membrane is excised.

The third step is to start making the diamond-shape anastomosis from the distal end of the distal duodenum halfway down the lower end of the bulbus with standing Vicryl 5/0 sutures. From there the anastomosis is continued distally toward the distal corner of the bulbus and then forward toward the proximal corner of the bulbus.

**Figure 5:**
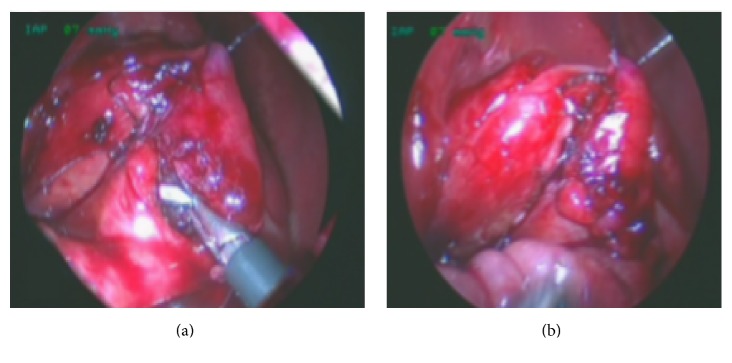
Diamond-shape anastomosis. (a) A nasoduodenal tube is inserted and pulled through the anastomosis under vision. (b) Completed anastomosis.

The nasoduodenal tube previously inserted is pulled through the anastomosis under vision and in case of doubts of distal obstruction saline is injected to test the canalization; finally, the ventral part of the anastomosis is laid to complete it, Figures 5(a) and 5(b). In Group 1 all the duodenoduodenostomies were performed with single interrupted stitches. In Group 2 we use either separate two running sutures for the posterior or anterior wall or single interrupted stitches without differences in the results. The choice of the suture was made considering the size of the surgical field in which we have performed the duodenal anastomoses trying to use the most ergonomic technique. In three cases we used an interrupted suture while in four we used two running sutures for the posterior or anterior wall of the duodenum. The colon is laid back over the duodenum and the trocars are removed under direct vision.

## 3. Results and Discussion

The demographics of the two groups were comparable. Patients median weight was 2742 g in Group 1 and 2495 g in Group 2. There was a prenatal diagnosis in both groups based on polyhydramnios and the detection of the double bubble sign, except for 3 patients in Group 1 and 1 in Group 2 who presented a prenatal diagnosis for oesophageal atresia. The male/female ratio was 3/7 in Open Group and 2/6 in MIS Group. The mean age was 36 weeks for both. The obstruction was preampullary in 9/10 patients in the Group 1 and in all 8 patients of Group 2. Multiple associated anomalies were seen in our patients including trisomy 21, cardiac anomalies, anorectal malformations (cloaca), pancreatic anomalies, laryngeal stenosis, and other intestinal malformations as oesophageal atresia and malrotation. Trisomy 21 was the most common anomaly, found in 6 of our patients (33%). Four patients were born prematurely (25%).

Most of our patients underwent surgery during the first week of life (Group 1 range: 1–26 days; Group 2: 1–4 days). All patients in both groups with intrinsic and extrinsic obstruction underwent diamond-shaped duodenoduodenostomy except the patient with duodenal web in Group 2 who was treated with endoscopic excision of the web. Hospital stay was 25 days for the Open Group and of 13-14 days for the MIS Group. The canalization was registered after an average of 8–12 days in Group 1 and 3 days in Group 2. Time to initiation of feeds averaged 3–5 days for laparoscopic procedures and 10–22 days for open procedures and time to full feeds averaged 7–9 days and 15–25 days, respectively.

A transanastomotic-tube was left in all patients of Group 1. It was used to start feeding and removed after 10–22 days (time to initiation of feeds). In Group 2 the nasoduodenal tube previously inserted is pulled through the anastomosis under vision and after the ventral part of the anastomosis was completed it was retired and positioned in the stomach (nasogastric tube). Only in the first patients was it used as TAT. In this case it was retired after 5 days and used as nasogastric tube. In Group 2 as in the Open one the tube was used for feeding. We did not record a delay in gastric emptying due to occlusion of the lumen in MIS Group patient. In Group 1 we had recorded a longer time to initial feeding and time to full oral intake with a slower reduction of daily volume of the fluid returned from the nasogastric (NG) tube which was bilious in the first days.

In MIS Group all cases were completed laparoscopically, and there were no intraoperative complications. The laparoscopic procedures were performed by the senior surgeon and there were no postoperative leaks, no missed distal intestinal obstructions, and no short-term/long-term complications. Postoperative UGI has been obtained in all cases.

In Group 1 (Open) malrotation was found in 2 patients and cloaca in another one; in Group 2 (MIS) we did not find malrotation, and we had oesophageal atresia associated as gastrointestinal malformation in one patients. With malrotation Ladd's procedure was performed without particular difficulty. In these series we did not perform Ladd's procedure laparoscopically, but we have experience in our centre of this procedure in mininvasive surgery.

Comparing the two groups average operating time was 120 min in Group 1 and 180–240 min in Group 2. Operative time obtained was that recorded by the scrub nurse and anaesthesiologist from initial operative start time to final skin closure. Detailed data on operative time for the laparoscopic duodenoduodenostomy alone (i.e., excluding time for additional procedures) were not available in all cases. The length of postoperative hospitalization, time to initial feeding, and time to full oral intake were all statistically shorter in patients undergoing a laparoscopic repair, Table 2.

Newborn and infants may require a laparotomy for a wide variety of intra-abdominal conditions. Surgeons traditionally have used open approach to address these conditions, but recent advances in mininvasive surgical techniques have kindled an interest in a minimally invasive approach to a wide variety of abdominal pathologies. These laparoscopic procedures have been shown to be technically possible, equally efficacious, and cosmetically superior.

Duodenal obstruction, such as that resulting from atresia or web, is one of these conditions which routinely has been corrected by laparotomy and duodenoduodenostomy. Atresia is classified into intrinsic and extrinsic form. The intrinsic atresia includes the following (Gray and Skandalakis): Type I (92%) with a web formed by mucosa and submucosa and an intact mesentery; this type includes the possible variant of windsock deformity (the membrane is thin and elongated); Type II (1%): two blind ends of duodenum connected by a fibrous short cord with intact mesentery; Type III (7%): the 2 blind ends being completely separated with a V-shaped mesentery defect. The extrinsic forms are prevalently represented by annular pancreas and Ladd's bands.

The first report of surgical correction of DA was by Ladd in 1931 with a reported mortality of 40% [7]. Over the last decades the improvements in operative techniques and postoperative care and the advancements in neonatal intensive care, parenteral nutrition, and management of associated anomalies have reduced mortality to 5–10%, related mostly to important heart malformations [1]. Several techniques have been described for the repair of duodenal atresia. Prior to the mid-1970s duodenojejunostomy was the preferred technique followed by side-to-side duodenoduodenostomy, partial web resection with Heineke-Mikulicz type duodenoplasty, and tapering duodenoplasty. The diamond-shaped duodenoduodenostomy described by Kimura et al. in 1990 [8] has become the standard. Recent improvements in laparoscopic equipment and techniques have sparked a revolution in the surgical care of infants and children. The introduction of advanced laparoscopic techniques in the neonate has more recently led to a new surgical approach, the laparoscopic duodenoduodenostomy [1]. The first reports of laparoscopic repair of duodenal atresia date 2001 and 2002, when shortly after each other Bax et al. [9] and Rothenberg [10] described their initial experience with this approach [11]. We revisited the patients with DA treated in the last 10 years comparing the open and the minimally invasive (MIS) approach describing our early experience with laparoscopic duodenoduodenostomy.

The application of MIS for the correction of congenital anomalies has increased significantly over the last years. The ability to perform delicate dissection and intracorporeal anastomosis has enlarged the scope of entities that can be approached. Although most neonatal conditions presenting with bowel obstruction present a difficult problem for laparoscopy because of the dilated bowel and limited abdominal cavity, this is not the case in duodenal atresia. In these patients, the entire small and large bowel are decompressed, allowing for excellent workspace even in low birth babies (according to our surgical experience with neonatal MIS approach) and there is an excellent exposure of the proximal duodenum. The laparoscope helps achieve a magnification of the operatory intra-abdominal field and consequently an accurate anastomosis even in bowel with a diameter of less than 5 mm [12]. The lack of distal bowel manipulation and probably the most declivous anastomosis seems to result in a shorter ileus and earlier initiation of feeds as described in a recent report by Spilde et al. [13]. They compared the laparoscopic and open approach to congenital duodenal obstruction and showed significantly shorter time to initiation of feeds, time to full feeds, and postoperative hospitalization in their laparoscopic group.

According to our experience (not only limited to neonate with CDO) and regarding patient outcomes, we found that the laparoscopic approach for CDO repair resulted in significantly shorter postoperative hospitalization, shorter time to initial feeding, and a shorter time to full oral intake. Comparing laparoscopic and open procedures (as suggested by multiple authors) these reductions may be attributed to less inhibition of bowel function and an abbreviated ileus related to the laparoscopic approach when compared to the open operation [13, 14]. We also recorded in MIS Group, compared with Open Group, a faster reduction of daily volume of the fluid returned from the nasogastric (NG) tube, which was no longer bilious in nature. We considered this sign a direct indication of an abbreviated ileus. Moreover, the postoperative UGI contrast studies, used routinely to evaluate for anastomotic leaks, help in this management leading us to remove the NG tubes after the contrast study showing no leak and contrast progression through the anastomosis. In this report, the mean time to NG tube removal for the laparoscopic group was 5 days with initial feeding start at 6–12 hours later. However, the latest cases treated showed an anticipation of the beginning of nutrition in the third postoperative day, also before the radiological study.

One reported disadvantage of the laparoscopic approach, as described in the reports following the first of Rothenberg in 2002, was the postoperative leak rate after conventional suturing techniques, considered unacceptable. For this reason, the U-clips were introduced to perform the anastomosis laparoscopically [1, 2]. All of our cases, 7 have been performed as described also by Kay et al. [1] with conventional suturing techniques without any observed leaks using both a running and interrupted suture line without complication. During the procedure we mobilize the second and third portions of the duodenum adequately using a “no touch” technique as much as possible to allow a tension-free diamond-shaped duodenoduodenostomy, reducing the risk of leakage.

**Figure 6:**
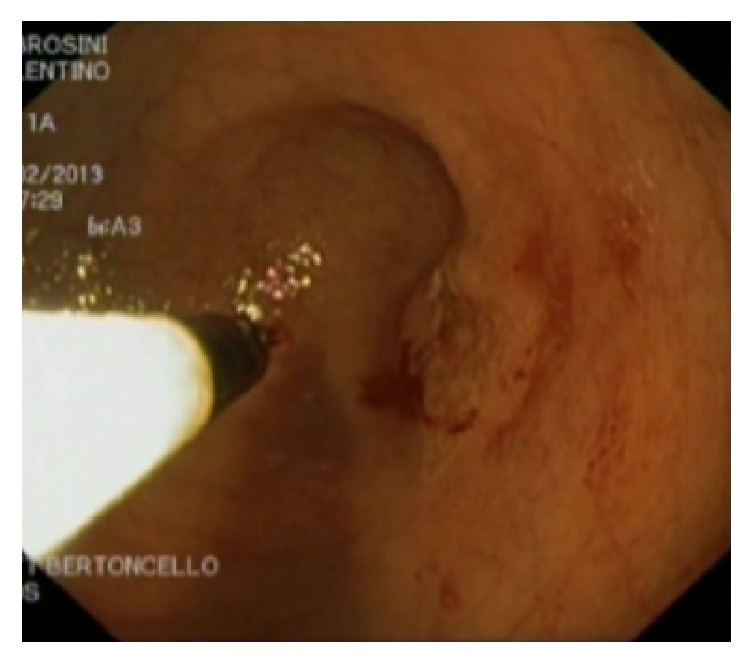
Endoscopic resection of duodenal web.

In our series, we included one patient with a clear duodenal web (associated with oesophageal atresia). In this patient, the diagnosis of duodenal obstruction was delayed after thoracoscopic repair of oesophageal atresia and underwent endoscopic resection of the web [15], Figure 6.

Another possible disadvantage of mininvasive approach is the difficult evaluation of the distal bowel to diagnose other associated intestinal atresia. During open procedures traditionally it is mandatory to inspect visually the bowel for distal atresic/obstructed segment. In this way, internal webs are more difficult to see. When the doubt of a web is high, the practice is to infuse the bowel with saline solution to confirm or not the obstruction. What we can affirm, according to what is described by other reports, is that despite the reduced possibility of detecting distal atresia (extremely low, <2%) [3, 16, 17], if time is taken laparoscopically to run the bowel, which is decompressed, only Type I atresia (web) could be missed. To infuse the bowel with saline is more difficult to perform laparoscopically (we do not perform it routinely) but we considered that the advantages of MIS approach are greater and more significant than this small risk. In our study we in both the laparoscopic and open approaches we did not record any incidence of bleeding, need of conversions or leakage; only one case of stricture formation in Open Group. The follow-up of our series for laparoscopic group ranges from 6 years to 6 months. This group, according to us, does not represent a large series, but the rarity of congenital anomaly (CDO) is such that allows us to affirm the effectiveness of laparoscopic procedure despite reports which described high leakage rate and other complications. Our results suggest that laparoscopic duodenoduodenostomy is safe and effective in surgeons with adequate laparoscopic skills.

## 4. Conclusions

Nowadays the prognosis for patient with CDO is excellent. Laparoscopic repair of duodenal atresia is a very elegant technique to restore continuity of the duodenum. The patient seems to benefit from the laparoscopic approach, for quick recovery and early oral refeeding, which lead to a fast return to full oral nutrition and discharge, as we show in this series, comparing with the traditional approach. According to our experience, the bowel with this approach is exposed to fewer risks deriving from its exteriorization, exposure, and hydroelectrolytic losses and in terms of manipulation. In summary, our experience demonstrates that laparoscopic duodenoduodenostomy can be performed safely and successfully even in the neonate with excellent short-term outcomes. Obviously, what is possible to conclude by a revision of the reports of the literature and by our direct experience is that conditions (CDO and DA, like OA) required a very experienced pediatric endoscopic surgical group (composed of surgeon, anaesthesiologist, pediatrician, and nurse) with a high level of expertise.

## Figures and Tables

**(a):** 

	Type I	Type II	Type III	Extrinsic obstruction
2004–2009 *Group 1: open approach (n = 10)*	3	—	5	2
2009–2015 *Group 2: mininvasive approach (n = 8)*	1	—	5	2

**(b):** 

Associated congenital anomalies	Open group (1)	Mininvasive group (2)
Trisomy 21	3	3
Congenital heart disease	—	1
Gastrointestinal disease	3	1
Genitourinary	—	1
Airways disease	—	1

**Table 2:** Main outcome variables in the babies undergoing repair of CDO.

Outcome Variable	Open approach (*N* = 10)	Mininvasive approach (*N* = 8)
Operative time	120 min	180–240 min
Length of postoperative hospitalization	25 days	12–14 days
Canalization	8–12 days	3 days
Time to initial feeding	10–22 days	3–5 days
Time to full oral intake	15–25 days	7–9 days
UGI studies	8–15 days	4–7 days
Evidence of stricture	1	—
Leakage	—	—
